# Role of p90 ribosomal S6 kinase in long-term synaptic facilitation and enhanced neuronal excitability

**DOI:** 10.1038/s41598-020-57484-y

**Published:** 2020-01-17

**Authors:** Rong-Yu Liu, Yili Zhang, Paul Smolen, Leonard J. Cleary, John H. Byrne

**Affiliations:** Department of Neurobiology and Anatomy. W. M. Keck Center for the Neurobiology of Learning and Memory, McGovern Medical School at the University of Texas Health Science Center at Houston, 6431 Fannin Street, Suite MSB 7.046, Houston, TX 77030 USA

**Keywords:** Cellular neuroscience, Learning and memory, Molecular neuroscience, Synaptic plasticity, Synaptic transmission

## Abstract

Multiple kinases converge on the transcription factor cAMP response element-binding protein (CREB) to enhance the expression of proteins essential for long-term synaptic plasticity and memory. The p90 ribosomal S6 kinase (RSK) is one of these kinases, although its role is poorly understood. The present study exploited the technical advantages of the *Aplysia* sensorimotor culture system to examine the role of RSK in long-term synaptic facilitation (LTF) and long-term enhancement of neuronal excitability (LTEE), two correlates of long-term memory (LTM). Inhibition of RSK expression or RSK activity both significantly reduced CREB1 phosphorylation, LTF, and LTEE, suggesting RSK is required for learning-related synaptic plasticity and enhancement in neuronal excitability. In addition, knock down of RSK by RNAi in *Aplysia* sensory neurons impairs LTF, suggesting that this may be a useful single-cell system to study aspects of defective synaptic plasticity in Coffin-Lowry Syndrome (CLS), a cognitive disorder that is caused by mutations in *rsk2* and associated with deficits in learning and memory. We found that the impairments in LTF and LTEE can be rescued by a computationally designed spaced training protocol, which was previously demonstrated to augment normal LTF and LTM.

## Introduction

In mammals, gene deletion studies have shown an essential role for the *rsk2* gene in cognitive functions^1^. In several animal models, the p90 isoform of RSK, expressed from *rsk2*, phosphorylates (i.e., activates) CREB after activation by the ERK isoform of MAP kinase (MAPK)^2–6^. Coffin-Lowry Syndrome (CLS) is caused by X-linked mutations in *rsk2*, and is associated with human intellectual disability and skeletal abnormalities^7^. A mouse *rsk2*-null model is deficient in fear memory consolidation and stimulus-induced CREB phosphorylation^1,8^. CREB-mediated transcription is required for memory formation in vertebrates and invertebrates. In mammalian systems, many of the signaling pathways that lead to phosphorylation of CREB have been well characterized, including the cAMP/PKA pathway and the Ras/ERK pathway^9^. However, the potential ERK/RSK/CREB pathway has received little attention as a potential contributor to synaptic plasticity, including long-term potentiation (LTP) and long-term synaptic facilitation (LTF), and also to long-term enhancement of neuronal excitability (LTEE). LTP, LTF, and LTEE are known correlates of long-term memory (LTM). To our knowledge, no study so far has examined the effects of RSK inhibitors on synaptic plasticity or excitability. One study examined the role of RSK2 in LTP using a RSK2 mutant mouse^8^. A negative result was reported – LTP was normal in the mutant. The negative result may be explained by the effects of RSK activation not being expressed during the period of observation (120 minutes), or a different isoform of RSK may have compensated for the mutant. Consequently, the role of RSK in long-term synaptic plasticity and enhancement of excitability remains unsettled.

The present study exploited advantages of the *Aplysia* sensorimotor culture system to examine the role of RSK in LTF and LTEE. LTF can reliably be maintained for days after induction^10–14^. In addition, only one RSK isoform similar to vertebrate p90 RSK is present in *Aplysia*^15^ reducing the possibility of compensation by other isoforms.

## Results

### 5-HT induced phosphorylation of CREB1 was attenuated by MEK inhibition

**Figure 1:**
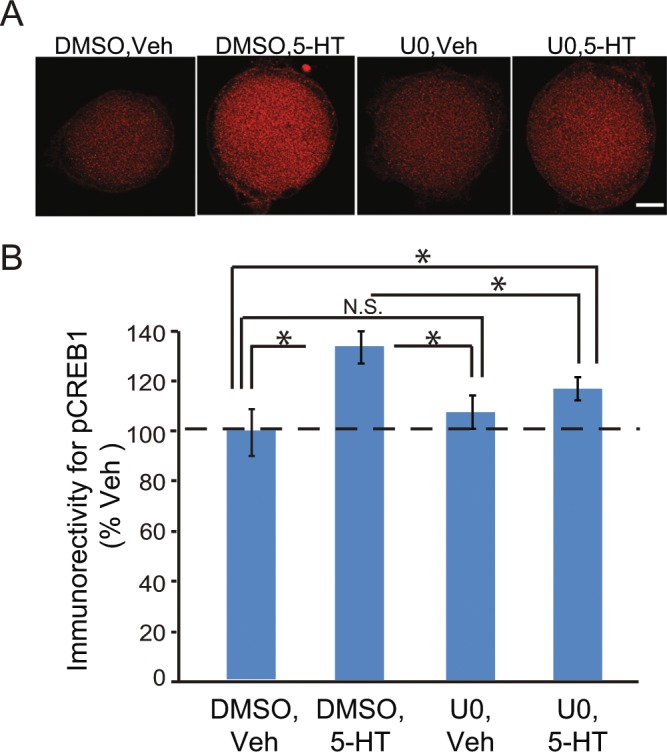
5-HT induced phosphorylation of CREB1 was attenuated by MEK inhibition. (**A**) Representative confocal images of pCREB1 immunofluorescence in SNs 2 h after 5 pulses of Veh or 5-HT. Immunoreactivity was located in the nucleus. Scale bar, 20 μm. (**B**) Summary data. 5-HT induced increase in pCREB1 was blocked by the MEK inhibitor U0126 (U0) (n = 5 independent experiments and in each experiment 5–10 SNs were analyzed in each group). In this and subsequent illustrations, significant differences are indicated by * for *P* < 0.05, and N.S. indicates that the difference between two groups is not significant.

We first examined whether 5-HT-induced phosphorylation (i.e., activation) of CREB1 was affected by blocking ERK activation. Sensory neurons (SNs) were pre-incubated for 0.5 h with 20 μM U0126 (U0), a MEK inhibitor, or DMSO control, followed by the Standard protocol of 5-HT. The Standard protocol is treatment of SN cultures with five, 5-min pulses of 50 µM 5-HT with a uniform interstimulus interval (ISI) of 20 min (onset to onset)^10^, and is commonly used to induce LTF (see Materials and Methods). SNs were fixed 2 h after 5-HT, a time when a significant increase in pCREB1 is observed, and processed for immunofluorescence^16^. Compared to DMSO + Veh, DMSO + 5-HT led to a 33 ± 6% increase in pCREB1, whereas the increase with U0 + 5-HT was 17 ± 5% (Fig. 1). The U0 + Veh alone group showed a slight increase in pCREB1 (9 ± 7%). A one-way repeated measures (RM) ANOVA on the raw data revealed a significant overall effect of the treatments. (*F*_*3,11*_ = 13.68, *P* < 0.001). Subsequent pairwise comparisons revealed that, as expected, the increase in pCREB1 in the DMSO + 5-HT group was significantly different from the DMSO + Veh group (q = 5.51, *P* < 0.001). Moreover, there was a significant difference between the DMSO + 5-HT group and the U0 + 5-HT group (q = 4.35, *P* < 0.05), and the U0 + 5-HT group was significantly different from the DMSO + Veh group (q = 4.35, *P* < 0.05), indicating that the 5-HT-induced phosphorylation of CREB1 is only partially blocked by U0126. U0 itself did not significantly decrease the basal level of pCREB1 (U0 + Veh *vs*. DMSO + Veh, q = 1.90, *P* > 0.05).

### 5-HT-induced increases in phosphorylated RSK were blocked by a MEK inhibitor

**Figure 2:**
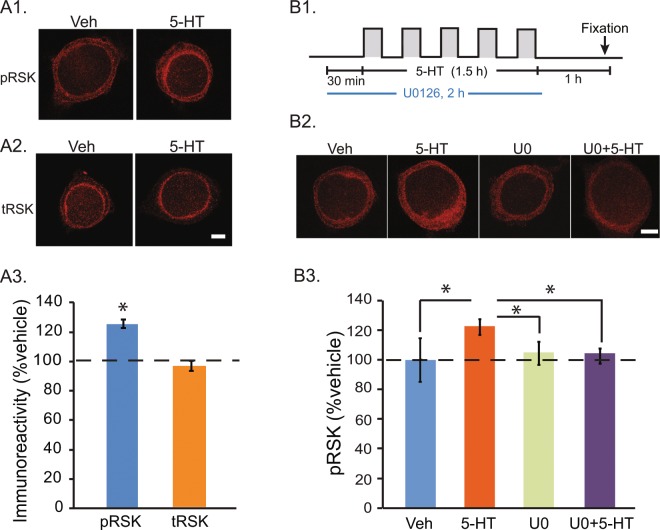
5-HT-induced increase in pRSK was blocked by a MEK inhibitor. (**A**) 5-HT increased pRSK immunoreactivity without affecting tRSK. (**A1**) Representative confocal images of pRSK immunofluorescence in SNs 1 h after 5 pulses of Veh or 5-HT. Immunoreactivity was located in both the cytoplasm and nucleus. (**A2**) Immunoreactivity for total RSK. (**A3**) Summary data. Significant differences between 5-HT and Veh are indicated by * for *P* < 0.05 (Paired t-test, n = 5 independent experiments). (**B**) Immunoreactivity for pRSK after 5-HT was blocked by U0126 (U0). (**B1**) Protocol for U0126 application with 50 μM 5-HT or Veh. (**B2**) Representative confocal images of pRSK in SNs 1 h after treatment. (**B3**) Summary data. 5-HT-induced increase in pRSK was blocked by preincubation with U0 (n = 4 independent experiments). Scale bar, 20 μm.

The above data suggest the MEK/ERK pathway is involved in the activation of CREB1. Given that mammalian p90 RSK2 is activated by MAPK and in turn phosphorylates CREB both *in vitro* and *in vivo*^2^, it is plausible that in *Aplysia*, ERK-dependent CREB1 phosphorylation is mediated by an RSK2 homolog. We located a predicted p90 RSK protein in the *Aplysia* genome (Accession ID: XM_005094731) with multiple ERK1/2 phosphorylation sites (Fig. S1A). To study the RSK cascade in *Aplysia*, we measured total levels of RSK (tRSK) and separately measured phosphorylated RSK [pRSK (residue Thr573)] using commercially available antibodies (see Methods). To assess the specificity of these antibodies, whole cell extracts of *Aplysia* nervous system were prepared and Western blot analysis was performed following established procedures^14^. Both antibodies recognized a single band with a molecular weight of ~90 kDa (Fig. S1B), which is consistent with the expected size of *Aplysia* p90 RSK. We next examined whether RSK activity is regulated by 5-HT treatment. SNs were exposed to the Standard 5-HT protocol or vehicle treatment, and were fixed for immunofluorescence 1 h later. We selected the 1 h time point because a significant increase in pCREB1 was detected 2 h after 5-HT treatment^16^, and we assumed RSK would be activated at an earlier time point. Compared to Veh, 5-HT led to a 25 ± 8% increase in pRSK (paired t-test using raw data, *t*_4_ = 2.99, *P* = 0.04), whereas no significant difference was found in total RSK protein levels (1 ± 3.5%, *t*_3_ = 0.15, *P* = 0.89) (Fig. 2A).

Next, we examined whether the 5-HT-induced upregulation of pRSK is mediated by the ERK pathway. Compared to DMSO + Veh, DMSO + 5-HT led to a 23 ± 6% increase in pRSK, whereas the increase with U0 + 5-HT was 6 ± 5%, which was similar to the U0 + Veh alone group (6 ± 8%)(Fig. 2B). A one way RM ANOVA revealed a significant overall effect of the treatments (*F*_*3,9*_ = 5.23, *P* = 0.02). Subsequent pairwise comparisons showed that, as expected, the increase in pRSK in the DMSO + 5-HT group was significantly different from the DMSO + Veh group (q = 5.26, *P* = 0.02). The U0 + 5-HT group was not significantly different from the DMSO + Veh group (q = 1.05, *P* = 0.48), and there was a significant difference between the DMSO + 5-HT group and the U0 + 5-HT group (q = 4.2, *P* = 0.04). These data indicate the 5-HT-induced phosphorylation of RSK is blocked by U0126. Notably, U0 itself did not significantly change basal levels of pRSK (U0 + Veh vs. DMSO + Veh, q = 1.53, *P* = 0.54).

### The Standard 5-HT protocol induced complex dynamics of pRSK

**Figure 3:**
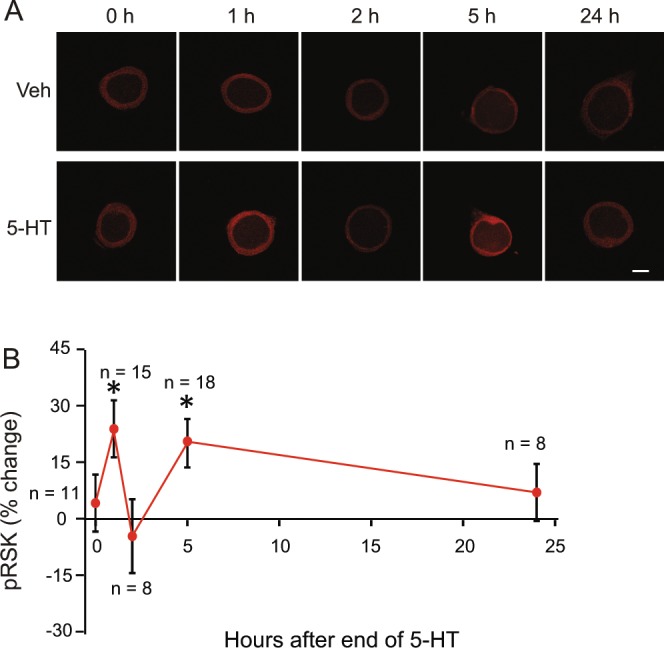
Biphasic regulation of pRSK by the Standard protocol (five 5-min pulses of 5-HT, 50 μM). (**A**) Representative confocal images of pRSK immunofluorescence in SNs at different times after the end of 5-HT. Scale bar, 20 μm. (**B**) Summary data. The percent change was calculated as the change of pRSK level after 5-HT compared to the control level. pRSK was elevated at 1 h after treatment, followed by a decrease at 2 h. A second wave of increase was evident at 5 h, and pRSK levels returned to basal levels at 24 h. Statistical analysis (Wilcoxon signed rank test with Bonferroni correction) revealed significant differences between Veh and 5-HT treatment groups at 1 h and 5 h.

We characterized the dynamics of pRSK during formation of LTF. SNs were treated with the Standard protocol of either vehicle or 5-HT, and then fixed at the time points corresponding to those previously used to investigate CREB1 and CREB2 dynamics after 5-HT^13,14^. Compared to time- matched controls, 5-HT increased pRSK at 1 h (24.4 ± 7.7%, n = 15) (Fig. 3). This initial increase was transient because pRSK quickly returned to baseline at 2 h (−4.5 ± 9.8%, n = 8). A second increase was detected at 5 h (20.2 ± 5.7%, n = 18), and this increase eventually returned to baseline at 24 h (7.1 ± 7.6%, n = 8). Statistical analyses using Bonferroni corrections for multiple comparisons of independent measurements revealed that the increases at 1 h and 5 h were both significant compared to control (at 1 h, Z = 2.84, *P* = 0.015; at 5 h, Z = 3.20, *P* < 0.005), whereas those immediately (4.2 ± 7.5%, Z = 0.45, *P* > 3), at 2 h (Z = −0.42, *P* > 3), and at 24 h (Z  = 0.84, *P* > 2) were not. These results indicate that the Standard protocol leads to a complex regulation of pRSK; an initial increase, followed by a decline, then a second wave of increase.

### Pharmacological inhibition of RSK reduced the 5-HT-induced phosphorylation of CREB1 and reduced LTF

**Figure 4:**
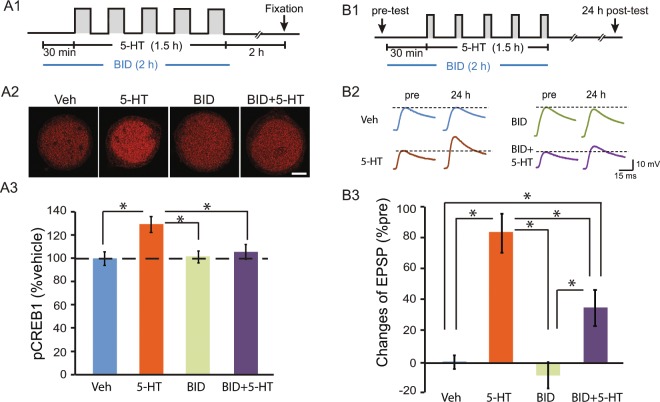
Inhibition of RSK reduced the 5-HT-induced phosphorylation of CREB1 and reduced LTF. (**A**) BID, a RSK inhibitor, blocked 5-HT-induced increases in pCREB1. (**A1**) Protocol for BID application with 5-HT or Veh. (**A2**) Representative confocal images of pCREB1 in SNs 2 h after treatment. (**A3**) Summary data. 5-HT-induced increase in pCREB1 was blocked by pre-incubation with BID (n = 6 independent experiments). Scale bar, 20 μm. (**B**) BID attenuated LTF. (**B1**) Protocol for BID application with 5-HT or Veh and EPSP recording. (**B2**) Representative EPSPs recorded immediately before (pre) and 24 h after treatment with 5-HT or Veh. Dashed lines represent the amplitude of the pre-test EPSPs. (**B3**) Summary data. Application of BID significantly reduced LTF produced by the S protocol (Veh, n = 7; 5-HT, n = 8; BID, n = 7; BID + 5-HT, n = 7).

Based on these results and on the reported phosphorylation of mammalian CREB by RSK, we hypothesized that the MEK/ERK pathway contributes to synaptic plasticity through activation of RSK. To test this, we applied the RSK1/2/3 inhibitor BI-D 1870 (BID)^17^, and examined the effects on CREB1 phosphorylation and LTF. Compared to Veh, 5-HT led to a 28.6 ± 5.8% increase in pCREB1 (Fig. 4), The increase in pCREB1 in the 5-HT + BID group was 5.4 ± 5.1% of control, and in the BID alone group 0.9 ± 4.3% of control (One-way RM ANOVA, *F*_*3,15*_ = 9.61, *P* *<* 0.001). Post hoc comparisons (SNK) indicated that pCREB1 was significantly elevated by 5-HT alone (5-HT vs. Veh, q = 7.22, *P* < 0.001). However, 5-HT + BID, and BID alone, did not significantly increase pCREB1 compared to Veh (5-HT + BID vs. Veh, q = 2.56, *P* = 0.201; BID vs. Veh, q = 1.64, *P* = 0.27). A significant difference between the 5-HT + BID group and 5-HT group (q = 4.67, *P* = 0.005), indicated BID blocked the 5-HT-induced elevation in pCREB1.

It is likely that this selective block of CREB1 phosphorylation by BID would impair synaptic plasticity. To test this hypothesis, LTF was induced by the Standard 5-HT protocol (Fig. 4B1). 5-HT increased EPSP amplitude by 82.4 ± 11.3% (n = 8) 24 h after treatment (Fig. 4B), but by only 34.9 ± 11.6% (n = 7) in the 5-HT + BID group (One-way ANOVA; F_3,25_ = 20.70, *P* < 0.001). Post hoc comparisons of the main effects revealed 5-HT alone significantly induced LTF (5-HT vs. Veh, q = 8.85, *P* = 0.001). RSK inhibition by BID did not affect basal synaptic transmission (pre-test EPSP amplitude, BID vs. Veh, q = 1.17, *P* = 0.416). Although the combination of 5-HT with BID still induced significant LTF (5-HT + BID vs. Veh, q = 3.59, *P* = 0.018), the strength of LTF in the BID + 5-HT group is significantly weaker than in the 5-HT group (5-HT + BID vs. 5-HT, q = 5.13, *P* = 0.001), indicating that blocking RSK with BID only partially blocked 5-HT-induced LTF. However, in the presence of BID, 5-HT-induced phosphorylation of CREB1 was close to basal level (Fig. 4A3). BID alone did not significantly change the resting potential or input resistance of the motor neurons (MNs) (Fig. S2A,B).

### Knockdown of RSK by siRNA injection also reduced the 5-HT induced phosphorylation of CREB1 and reduced LTF

**Figure 5:**
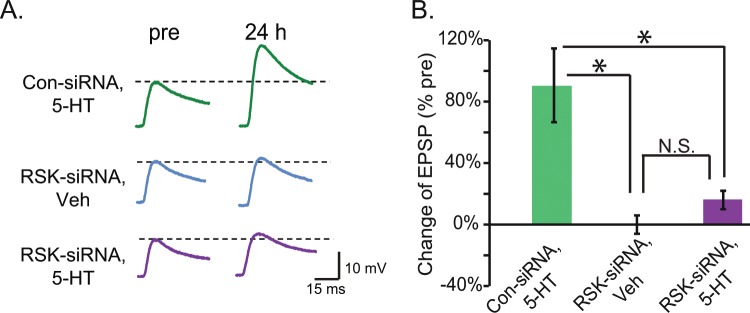
Knock down of the expression of RSK by siRNA injection reduced LTF. (**A**) Representative EPSPs recorded immediately before (pre-test) and 24 h after (post-test) treatment with 5-HT. Dashed lines represent the amplitude of the pre-test EPSP. (**B**) Summary data. RSK-siRNA significantly reduced 5-HT-induced LTF [(Con-siRNA, 5-HT), n= 6; (RSK-siRNA, Veh), n= 6; (RSK-siRNA, 5-HT), n = 6].

In Fig. 4, the RSK1/2/3 inhibitor BID was used to block *Aplysia* RSK signaling. Although not characterized yet, other forms of *Aplysia* RSK besides the RSK2 homolog may also be inhibited by BID. Therefore, to further examine the role of the *Aplysia* RSK2 homolog in phosphorylation of CREB1 and LTF, we used siRNA to reduce basal RSK expression (Fig. S3A). The Standard 5-HT-induced phosphorylation of CREB1 in SNs injected with RSK-siRNA averaged 28 ± 5% less than that in Con-siRNA injected, 5-HT-treated SNs (Fig. S3A, paired t-test, *t*_4_ = 4.48, *P* = 0.011). We next examined the effects of RSK knockdown on LTF using three groups of SN-MN co-cultures (Fig. 5). SNs from SN-MN co-cultures were injected with either RSK-siRNA or Con-siRNA. 96 h after injection, cultures were treated with the Standard protocol (RSK-siRNA + 5-HT, n = 6; Con-siRNA + 5-HT, n = 6) or vehicle control (RSK-siRNA + Veh, n = 6). Con-siRNA itself does not change basal synaptic strength^13,14,18,19^. Therefore, we omitted the Con-siRNA + Veh group in this experiment. In each group, EPSPs were measured immediately prior to the Standard protocol (pretest) and 24 h after treatment (post-test). One-way ANOVA indicated significant overall differences among the treatment groups (*F*_*2,15*_ = 9.914, *P* = 0.002). Post hoc comparisons (SNK) revealed a significant difference between the Con-siRNA + 5-HT group and the other two groups (Con-siRNA + 5-HT vs. RSK-siRNA + Veh, q = 5.96, *P* = 0.002; Con-siRNA + 5-HT vs. RSK-si RNA + 5-HT, q = 4.74, *P* = 0.005). Although the EPSP amplitude 24 h post-test increased by 18 ± 6% in the RSK-siRNA + 5-HT group, no significant difference was observed between the RSK-siRNA + Veh and RSK-siRNA + 5-HT groups (q = 1.2, *P* = 0.4). These results indicate that knockdown of RSK significantly attenuated LTF. This attenuation of LTF was not associated with differences among the pretest EPSPs of the Con-siRNA and RSK-siRNA groups (Paired t-test, *t*_16_ = 0.80, *P* = 0.438), suggesting RSK-siRNA had no effect on basal synaptic strength. These results demonstrate a previously underappreciated role for RSK in LTF.

### RSK siRNA-impaired LTF is restored by an Enhanced protocol

**Figure 6:**
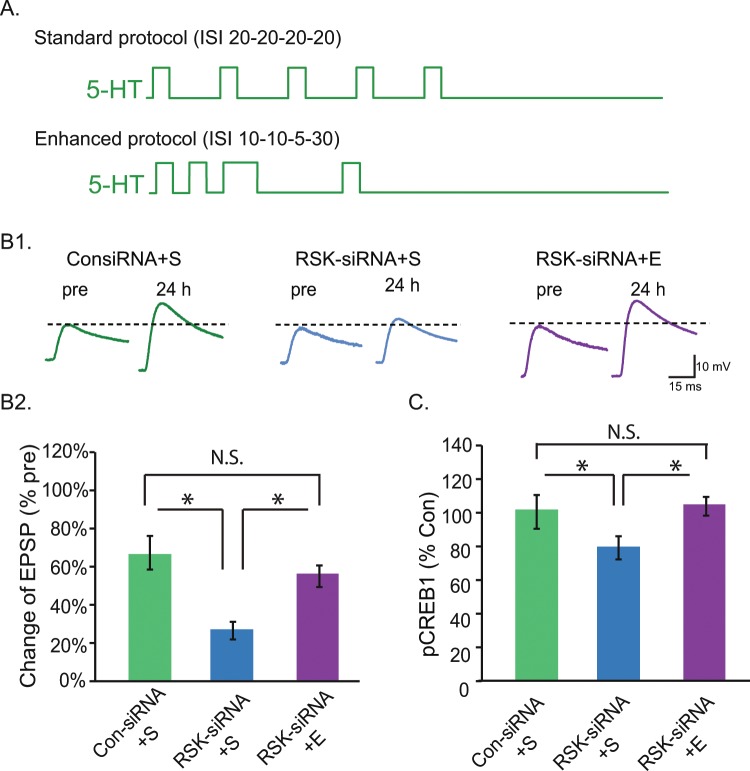
RSK-siRNA impaired LTF is restored by an Enhanced protocol. (**A**) Two 5-HT protocols. The patterns of 5-HT pulses are illustrated in each panel. The Standard protocol (S) had uniform ISIs of 20 min, whereas the Enhanced protocol (E) had non-uniform ISIs of 10, 10, 5 and 30 min. (**B**) The deficit in LTF induced by RSK-siRNA injection can be rescued by the Enhanced protocol. (**B1**) Representative EPSPs recorded immediately before (pre-test) and 24 h after (post-test) treatment with 5-HT or Veh. Dashed lines represent the amplitude of the pre-test EPSPs. (**B2**) Summary data. In each SN-MN pair, the EPSPs that were measured 24 h post 5-HT treatment were normalized to the pre-test EPSP. A value of 0% represents no change in EPSP amplitude. RSK-siRNA injection reduced S protocol-induced LTF, whereas this attenuation in EPSP amplitude was significantly rescued by the E protocol (Con-siRNA + S, n = 7; RSK-siRNA + S, n = 7; RSK-siRNA + E, n = 7). (**C**) The decreased pCREB1 in RSK siRNA-injected, S treated SNs was restored by the E protocol (Con-siRNA + S, n = 5; RSK-siRNA + S, n = 4; RSK-siRNA + E, n = 4).

An Enhanced protocol with irregularly spaced 5-HT applications (Methods) enhances the overlap of PKA and ERK activities upstream of CREB1 and CREB2, and increases LTF and LTM^10,20^. We hypothesized that this Enhanced (E) protocol may compensate for the effect of RSK knockdown by its augmentation of PKA/ERK interaction, thus normalizing the pCREB1 and LTF deficits, at least in part. To test this hypothesis, three groups of SN-MN co-cultures were treated with 5-HT (Fig. 6). In this experiment, we omitted the E alone control group, because we previously determined that the E protocol induced nearly twice the magnitude of LTF induced by the S protocol (average EPSP amplitude increase of 72% for E vs. 40% for S^10^, and the E protocol also significantly augmented the increase in pCREB1^10,21^). One way ANOVA indicated significant overall differences among the groups (Fig. 6B2) (*F*_*2,16*_ = 9.40, *P* = 0.002). Replicating the knockdown results of Fig. 5, LTF in the RSK-siRNA + S group (n = 5) was significantly less than that in the Con-siRNA + S group (n = 7, q = 6.02, *P* = 0.002). Importantly, LTF in the RSK-siRNA + E group (n = 7) was significantly greater than in the RSK-siRNA + S group (q = 4.46, *P* = 0.006). Moreover, no significant difference in LTF was observed between the Con-siRNA + S and RSK-siRNA + E groups (q = 1.71, *P* = 0.246). Therefore, the Enhanced protocol compensated for the deficit of RSK and restored LTF. This rescue of LTF was not associated with differences between the pretest EPSPs of the Con-siRNA and RSK-siRNA groups (one way ANOVA, *F*_*2,16*_ = 2.05, *P* = 0.161).

The Enhanced protocol may compensate for the deficit in pCREB1 induced by RSK knockdown. To test the effect of the Enhanced protocol on pCREB1, the same groups of SN cultures were treated with 5-HT as described above: Con-siRNA + S, RSK-siRNA + S, and RSK-siRNA + E. siRNA was injected into SNs 96 h before 5-HT and the immunoreactivity to pCREB1 was measured 2 h after the end of 5-HT (Fig. 6C). A one way RM ANOVA indicated significant overall differences among the groups (*F*_*2,7*_ = 15.01, *P* = 0.003). Replicating the knockdown results of Fig. S3B, immunoreactivity of pCREB1 in the RSK-siRNA + S group was significantly less than that of the Con-siRNA + S group (q = 6.21, *P* = 0.003). Importantly, pCREB1 levels in the RSK-siRNA + E group were significantly greater than that of the RSK-siRNA + S group (q = 7.24, *P* = 0.004). No significant difference in pCREB1 was observed between the Con-siRNA + S and RSK-siRNA + E groups (q = 1.03, *P* = 0.49). These results indicate that the Enhanced protocol compensated for the deficit of pCREB1. Consistent with BID effects on pCREB1 (Fig. 4), RSK-siRNA significantly diminished the elevation in pCREB1 induced by the Standard protocol (Fig. 6C). However, the Standard protocol still appeared to induce LTF in the RSK-siRNA group (Fig. 6B2).

### Pharmacological inhibition of RSK activity reduced 5-HT-induced LTEE and this impairment was rescued by the Enhanced protocol

**Figure 7:**
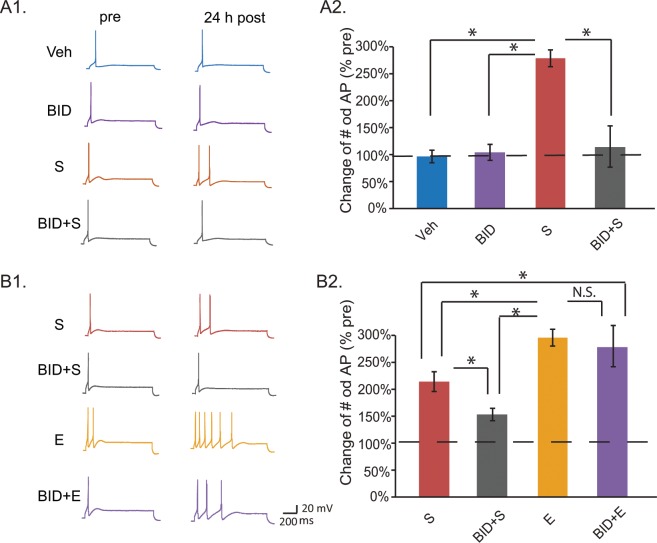
Enhanced training protocol rescued BID-induced impairment of LTEE. (**A**) BID blocked the S protocol-induced LTEE in isolated SNs. (**A1**) Action potentials were recorded from cultured SNs before (pre), and 24 h after treatment with S or vehicle (post). (**A2**) Summary data (24 h post-test). A value of 100% represented no change in cell excitability. A significant increase in cell excitability was found in the S group, but this LTEE was blocked by BID (Veh, n = 5; BID, n = 5; S, n = 6; BID + S, n = 5 independent experiments). (**B**) BID-blocked LTEE was restored by the E protocol. (**B1**) Electrophysiological recording of action potentials (APs). (**B2**) Summary data. The E protocol rescued BID-impaired LTEE (S, n = 8; BID + S, n = 8; E, n = 6; BID+E, n = 6 independent experiments).

In *Aplysia*, LTF is also accompanied by LTEE of SNs^13,22^. To determine the role of RSK in excitability, we used BID to specifically block the activity of RSK and then examined the effects on LTEE induced by the Standard 5-HT protocol. Excitability was assessed by counting the number of spikes elicited by a constant current pulse (Fig. 7) and was measured prior to 5-HT treatment (pre-test), and 24 h after treatment (post-test) in four groups: (1) Veh, (2) 5-HT (Standard protocol), (3) BID alone, and (4) BID + 5-HT (Fig. 7A). A significant interaction between groups was found at 24 h after treatment (Fig. 7A, averaged % pre ± SEM: Veh: 90 ± 11%, n = 5; 5-HT alone: 263 ± 14%, n = 6; BID alone: 97 ± 13%, n = 5; BID + 5HT: 106 ± 35%; one-way ANOVA, *F*_*3,17*_ = 18.04, *P* < 0.001). Subsequent pair-wise comparisons revealed that 5-HT produced a significant increase in the number of spikes compared to vehicle treatment (q = 8.67, *P* < 0.001), BID alone (q = 8.32, *P* < 0.001) or BID + 5HT (q = 7.86, p < 0.001). Importantly, the BID + 5-HT group was not significantly different from Veh (q = 0.80, *P* = 0.839), suggesting inhibition of the activity of RSK by BID impaired LTEE at 24 h. BID alone did not significantly alter the resting potential or input resistance of isolated SNs (Fig. S2C,D).

If LTF and LTEE have common control pathways, we would expect the Enhanced protocol to rescue the impairment of LTEE by BID. To test this hypothesis, SNs were treated with different 5-HT protocols in the presence of BID (Fig. 7B). LTEE induced by the Enhanced protocol (E, n = 6) was significantly greater than that induced by the Standard protocol (S, n = 8) (q = 4.50, *P* = 0.011). Moreover, consistent with the knockdown results of Fig. 6B, LTEE in = the BID + S group (n = 8) was significantly less than that in the S group (q = 3.89, *P* = 0.011). Importantly, LTEE in the BID + E group (n = 6) was significantly greater than in the BID + S group (q = 7.37, *P* <0.001) and greater than in the S group (q = 3.77, *P* = 0.014). In addition, no significant difference in LTEEs was observed between the E and BID + E groups (q = 0.68, *P* = 0.633). Therefore, the Enhanced protocol rescued the BID-induced impairment in LTEE.

## Discussion

### Role of RSK in long-term synaptic plasticity

The present study exploited the technical advantages of the *Aplysia* sensorimotor culture system to examine the roles of RSK in long-term synaptic plasticity and neural excitability. We found that a Standard LTF-inducing protocol leads to increased active RSK (pRSK) and CREB1 (pCREB1) in sensory neurons, that pRSK can phosphorylate and activate CREB1, and that the increases in pRSK and pCREB1 are blocked by a MEK inhibitor. Also, RSK siRNA reduced LTF measured 24 h after induction, and an inhibitor of RSK blocked 24-h LTF, suggesting a critical role of the ERK/RSK/CREB1 pathway in LTF. Basal synaptic transmission was not affected by RSK inhibition or RSK siRNA. This last result does contrast with the effect of constitutive *rsk* knockout in mouse (reduction of basal synaptic transmission induced by knockout of *rsk2*^8^), and in *Drosophila* (requirement of a RSK orthologue for normal synaptic morphology and function^23^). This could be due to the transient (only a few days) and partial knockdown of RSK (Fig. S3A, 28% decrease in RSK expression) in our system compared to the complete and lifetime effects of a genetic knockdown.

The reduction in RSK level and in LTF after RSK siRNA injection suggests *Aplysia* sensory neurons are a useful single-cell system for studying the impairment of synaptic plasticity and learning associated with CLS. We found, in addition, that impairments in both LTF and LTEE due to RSK inhibition were rescued by a computationally designed training protocol that augments normal LTF and LTM^10^. This result suggests that the strategy of combining computational models with empirical tests of model predictions, developed by our previous study^10^, may also be useful in mammals to predict optimized training protocols that enhance synaptic plasticity or rescue deficits in plasticity.

### Revised model of *Aplysia* LTF

**Figure 8:**
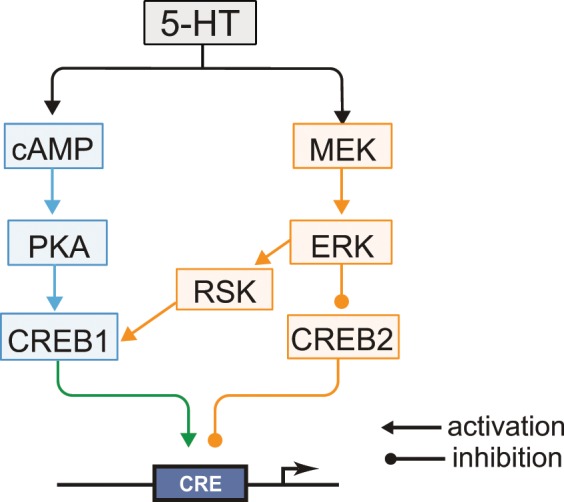
Schematic model describing activation of *Aplysia* CREB1, deactivation of *Aplysia* CREB2, and consequent induction of genes regulated by a cAMP response element (CRE) that is required for the induction of LTF. CREB1 can be phosphorylated by both PKA and RSK. Phosphorylation of CREB1 is assumed necessary for induction of gene expression. CREB2 represses gene expression. Phosphorylation of CREB2 by ERK removes this repression. Arrows and circles indicate positive and negative regulation of transcription, respectively.

This study also extends the understanding of mechanisms of LTF and LTEE in *Aplysia*. RSK has not been included in models of signaling pathways that mediate formation of LTF and LTM^24^. The prevailing view has been that activation of CREB1 occurs primarily through the PKA pathway^25^. The ERK MAPK pathway has been implicated in LTF, but with ERK phosphorylating the transcriptional repressor CREB2, removing CREB2’s inhibitory effect on genes regulated by CREB1^26,27^. Recent data has shown that ERK activates RSK2 and induces transcription of the immediate early gene *c/ebp* after one 5-min pulse of 5-HT^28^. That finding was intriguing, but did not demonstrate a role of RSK2 in LTF, because a 5-min treatment of 5-HT does not lead to LTF. We have substantially extended these results by confirming ERK activation of RSK, determining that phosphorylation of CREB1 is ERK-dependent, and demonstrating that this dependency is mediated by RSK. Inhibition of the ERK – RSK pathway by U0126, or of RSK by BID, partially blocked 5-HT-induced CREB1 phosphorylation (Fig. 1), indicating that the ERK – RSK pathway converges with the cAMP – PKA pathway to mediate CREB1 phosphorylation. Correspondingly, BID partially blocked 5-HT – induced LTF (Fig. 4). Inhibition of RSK expression by RSK siRNA reduced CREB1 phosphorylation/activation and 5-HT LTF, without affecting basal synaptic transmission (Figs. 1B, 4A and 5A). These results provide direct evidence for a previously underappreciated role for RSK as a CREB1 kinase in *Aplysia* as well as in LTF and suggest the revised model depicted in Fig. 8.

Intriguingly, the immunoreactivities for pERK and pRSK in *Aplysia* are mainly located in the cytoplasm. However, minor proportions of pERK^21,28–30^ and pRSK (Figs. 2 and 3) are detected in the nucleus. CREB1 is expressed in both cytoplasm and nucleus^13,14^. Therefore, it is possible that CREB1 can be phosphorylated by a minor extent by nuclear RSK. However, the immunoreactivity for pCREB1 is predominantly located in the nucleus. We do not have a definite explanation for why the ratio of nuclear pCREB1 to cytoplasmic pCREB1 appears to be substantially greater than the ratio of active nuclear pRSK to cytoplasmic pRSK. One possible explanation is that, subsequent to phosphorylation by cytoplasmic pRSK and nuclear translocation, pCREB1 becomes, to an extent, kinetically trapped in the nucleus. This nuclear localization would be due to enhanced binding of pCREB1 to *cre* elements that modulate expression of its numerous target genes.

### Dynamics of RSK

Activation of RSK exhibits complex dynamics that depend on the protocol of 5-HT treatment. It was previously reported^28^ that a single 5 min 5-HT treatment increased the phosphorylation of p90 RSK at 45 min post onset of treatment. This increase was no longer observed at 1 h. Here we extended the 5-HT treatment to the Standard protocol of five 5-min 5-HT pulses and examined multiple time points. There were two waves of RSK activation (Fig. 3), with peak elevations in phosphorylation of RSK at 1 h and at 5 h after 5-HT treatment, and less at 2 h. These long-lasting vs. transient dynamics of RSK in response to the different (5 pulses vs. 1 pulse) 5-HT protocols correspond respectively to the long-lasting vs. transient responses of PKA and ERK dynamics^28,29,31,32^. It will be important to further characterize the time courses of activation of the PKA and MAP kinase pathways, and how their interaction regulates CREB1 dynamics. Characterizing these dynamics will be important for understanding and predicting the efficacy of computationally designed training protocols to optimize LTF and LTM.

### Role of RSK in long-term enhancement of excitability

BID significantly impaired 5-HT-dependent LTEE (Fig. 7). To our knowledge, no previous study found a role of RSK in modulating the excitability of an individual characterized neuron, although a previous study showed modulation of excitability at the network level in *rsk2* knockout mice^8^. LTEE and LTF cooperate to induce long-term sensitization of withdrawal reflexes in *Aplysia* following aversive stimulation, and CREB1 plays important roles in both processes^13,33^. Similarly, in mammals, a learning-induced increase in CREB expression, and presumably activity, mediates enhancement in excitability, and therefore may contribute to neuron allocation into memory engrams as well as to memory consolidation^34–36^.

The E protocol yields substantially greater LTF, and higher pCREB1, than the S protocol^10^, whereas in the current study, we found that the E protocol together with RSK inhibition yields similar levels of LTF and pCREB1 as does the S protocol (Fig. 6). In combination these data imply that RSK inhibition would likely reduce LTF and pCREB1 in the E protocol, in contrast with the lack of effect of BID on LTEE induced by E (Fig. 7). A ceiling effect limiting the amount of LTEE that can be induced by the E protocol could explain the lack of effect of BID on LTEE produced by E alone. Alternately, the effect of RSK inhibition on LTEE induced by E may be compensated, in part, by a signaling pathway independent of RSK. One possibility is mitogen and stress-activated kinase (MSK). Although a MSK isoform has yet to be identified in *Aplysia*, MSK1 is an ERK-activated CREB kinase in vertebrates^37^. MSK could, if present in *Aplysia*, contribute to the phosphorylation of CREB1 or other substrates involved in regulating excitability.

### Rescue of deficits in LTF and LTEE

Previously, our laboratory designed a strategy to rescue deficits in LTF in a cellular model for deficits in synaptic plasticity in Rubinstein-Taybi syndrome (RTS)^16^, generated by inhibiting the expression of CREB-binding protein (CBP) in *Aplysia* SNs. A computationally designed, irregularly spaced training protocol, predicted to be effective by simulating the biochemical signaling pathways that converge on CREB1 and mediate LTF, was verified empirically to rescue deficits in LTF associated with the CBP knock down^16^. Similarly, our current results suggest that impairment of LTF due to knock down of RSK may serve as a cellular model for synaptic plasticity impairment in CLS^7^.

Using this model system, we have begun to investigate efficient novel treatments to rescue LTF deficits associated with RSK knockdown. The LTF deficit was rescued by the previously developed^10^ computationally designed Enhanced protocol (Fig. 6). In addition, the Enhanced protocol not only augmented 5-HT-induced LTEE, but also rescued the deficit in LTEE due to inhibition of RSK by BID (Fig. 7). Previous computational modeling (Zhang *et al*. 2012) suggested that the increased effectiveness of this irregularly spaced Enhanced (E) protocol is likely due, at least in part, to two factors: (1) the ability of the E protocol to increase the temporal overlap between activation of PKA and of ERK^10^, which would improve the coincidence of CREB1 phosphorylation by PKA with CREB2 phosphorylation by ERK, and (2) a higher ERK activation peak compared to the Standard protocol. In the present study, RSK was only partially knocked down by siRNA (Fig. S3A). In this case the increased ERK activation and consequent increased residual RSK activation produced by the Enhanced protocol, combined with the increased overlap of PKA and ERK activation, may compensate for the reduced RSK protein level, thereby restoring the normal increase in pCREB1 after 5-HT applications, and consequently rescuing LTF and LTEE.

Persistent LTEE and LTF after aversive stimulation in *Aplysia* might also constitute an invertebrate model of neuronal hyperarousal in post-traumatic stress disorder, or in mammalian neuropathic pain^38,39^. Given that the results presented here suggest RSK and CREB play key roles in both LTEE and LTF, studies may be warranted to investigate whether inhibiting RSK and consequent CREB phosphorylation ameliorates neuronal hyperarousal in these chronic conditions.

## Materials and Methods

### Animals and cell cultures

All experiments used primary cultures of identified cells from *Aplysia californica* (NIH *Aplysia* resource facility, University of Miami, Miami, FL). SNs were isolated from pleural ganglia of 60–100 gm animals, and MNs were isolated from abdominal ganglia of juvenile animals (0.8–1.5 gm). Animals were housed in plastic containers inside aerated tanks containing artificial seawater (ASW; Instant Ocean; Aquarium Systems) maintained at 15 °C. Isolated SNs or SN-MN co-cultures were prepared according to conventional procedures^10^. Dishes of SN cultures were plated with 5–10 SNs. Dishes of SN-MN co-cultures were plated with a single SN and a single L7 MN. Both isolated SN cultures and SN-MN co-cultures were allowed to grow at 18 °C, and the growth medium (containing 50% of adjusted L15 with 1.2 mg/ml of L-Glutamine and 50% of hemolymph) was replaced prior to treatments and recordings with a solution of 50% L15 and 50% artificial seawater (ASW; 450 mM NaCl, 10 mM KCl, 11 mM CaCl_2_, 29 mM MgCl_2_, 10 mM HEPES at pH 7.6)^10^.

### siRNA knockdown of RSK

A custom ON-TARGET plus SMART pool of RSK siRNAs was designed and provided by Dharmacon to target an *Aplysia* homolog of RSK, which has been used in previous studies^13,16,19^. Non-targeting siRNA was used as a negative control. Potential off-target effects were evaluated by examining biophysical properties (i.e., membrane resistance, resting membrane potential, cell excitability) to ensure the injections did not affect the health of the cells^13,16,19^. Before siRNA injection, cultures or co-cultures were allowed to grow for 2 d at 18 °C. siRNA (5 μM final concentration) was injected into SNs following established procedures^13,16,19^. Cultures and co-cultures were allowed to grow for another 4 d after siRNA injection at 18 °C. SN cultures were then processed for immunofluorescence and co-cultures were used for electrophysiological study.

### Pharmacological treatment

The Standard 5-HT protocol is to treat SN cultures or SN-MN co-cultures with five, 5 -min pulses of 50 µM 5-HT (Sigma) with a uniform interstimulus interval (ISI) of 20 min (onset to onset)^10^. The Enhanced protocol, in contrast, is comprised of five 5-min pulses of 50 µM 5-HT with ISIs of 10, 10, 5, and 30 min respectively^10^. A separate group of control cultures were not treated with 5-HT, but were treated with vehicle alone (Veh) (L15:ASW) with ISIs of the Standard protocol.

MEK is the upstream kinase of ERK. The MEK1/2 inhibitor U0126 (Cell Signaling) was used to block activation of the ERK pathway. At 20 μM, U0126 blocks ERK phosphorylation after 5-HT treatment in *Aplysia*^30,32,40^. U0126 was applied to SN cultures 30 min before and during 5-HT treatment.,

BI-D1870 (BID) is a small cell-permeant molecule that specifically inhibits mammalian RSK isoforms (RSK1 RSK2, RSK3 and RSK4) *in vitro* and *in vivo* by competitively inhibiting ATP at the N-terminal PKA/protein kin ase G/protein kinase C domain of RSK. At 10 μM, BID inhibits the phosphorylation of RSK substrates completely in mammalian cell lines^17^. The use of BID in *Aplysia* has not been reported. In a pilot study, we examined different concentrations of BID (1, 2, 5 and 10 µM) on phosphorylation of CREB1 (pCREB1), one of the RSK target proteins. We found that 5 and 10 µM BID tended to elevate basal pCREB1 levels in the absence of 5-HT treatment (increased by 13 ± 3.3%, n = 3). Therefore, to selectively study the contribution of RSK to 5-HT-induced changes in pCREB1 and LTF, we used 2 µM BID. SN or SN-MN co-cultures were exposed to BID for 30 min before, and throughout, the Standard or Enhanced protocol.

### Immunofluorescence

SNs were fixed for immunofluorescence at different times after 5-HT treatments as described in Results. Procedures followed those of Chin *et al*.^41^. In order to examine RSK levels after injection with control siRNA (Con-siRNA) or RSK-siRNA, cells were fixed 96 h after injection, without 5-HT treatment, in a solution of 4% paraformaldehyde in PBS containing 30% sucrose for 30 min. After three rinses in PBS, fixed cells were blocked for 30 min at room temperature in Superblock buffer (Pierce)/0.2% Triton X-100/3% normal goat serum and subsequently incubated overnight at 4 °C with antibodies against total p90 RSK (anti-tRSK, Cell Signaling, Cat # 9355, in 1:400 dilution), or phosphorylated p90 RSK [anti-pRSK (Thr573), Cell Signaling, Cat # 9346, also in 1:400 dilution], or against phosphorylated CREB1 (anti-pCREB1, 1:500)^42^. After primary antibody incubation, secondary antibody (goat anti-rabbit conjugated to Cy-3, Jackson Lab, 1:200 dilution) was applied to the cells for 1 h at room temperature. Cells were then mounted for confocal imaging analysis. The intensity of staining was quantified in images obtained with a Zeiss LSM510 confocal microscope using a 63x oil immersion lens as described previously^13^. A z-series of optical sections through the cell body (0.5 µm increments) was taken, and the section through the middle of the nucleus was used for analysis of mean fluorescence intensity of the whole cell with ImageJ-win64 software (NIH). All the neurons on each coverslip were analyzed, and measurements from these neurons were averaged. In each such experiment in Figs. 1, 2B, and 4A, four groups of SNs from the same animal were used for different treatments. These experiments were performed in a blind manner so that the investigator analyzing the images was unaware of the treatment the SNs received. The number of samples (n) reported in Results indicates numbers of dishes assessed.

To study the time course of RSK phosphorylation after the Standard 5-HT protocol (Fig. 3), dishes of SNs cultured from the same animals were paired for all of the 5-HT treatments. In each pair, one dish received L15:ASW as vehicle control (Veh). The other received the same solution with the addition of 50 μM 5-HT. The experimenter was blind to the identity of the treatments. During the ISIs of five pulses of 5-HT, SNs were incubated in L15/ASW after wash out of 5-HT. One pair of dishes was fixed immediately after the last wash out (i.e., 0 h). The other dishes were fixed 1, 2, 5, or 24 h later. The remaining dishes served as time-matched Veh controls. For each pair of dishes measured at the same time point, the averaged fluorescence intensity from the dish receiving 5-HT was compared to that from the Veh control. The number of samples (n) reported in the Results indicates the numbers of dishes assessed.

### Electrophysiology

Excitability of SNs was measured in a separate group of experiments. As we have previously described^13,41^, neurons were impaled with a single microelectrode (10–20 MΩ resistance) and current clamped at −45 mV. Input resistance was measured by applying 0.1 nA of hyperpolarizing current for 2 sec. Firing threshold was measured by applying 1 s of depolarizing current in increasing increments of 0.1 nA until an action potential was triggered. The lowest current intensity necessary to fire a single action potential was considered the firing threshold. Excitability was measured by counting the number of action potentials (APs) triggered by applying 0.5 nA of depolarizing current for 1 s. When the firing threshold of SNs was equal to or above 0.5 nA, but less than 1.0 nA, 1.0 nA of depolarizing current was used to measure excitability. The firing threshold for SNs is normally 0.3–0.7 nA. SNs were excluded from further use if cells had resting potentials more positive than −30 mV, failed to respond to depolarizing current up to 1.0 nA, or had a firing threshold less than 0.3 nA in the test prior to 5-HT treatment (pre-test). We recorded 3–5 neurons in each dish and measurements from SNs in the same dish were averaged. The number of samples (n) reported in the Results indicate numbers of dishes recorded. Treatment protocols and data analysis were similar to those described below for analysis of excitatory postsynaptic potentials (EPSPs).

EPSPs were recorded from MNs from the SN-MN co-cultures following established procedures^10,13,14,16,19^. Stimulation of presynaptic SNs was performed extracellularly using a blunt patch electrode filled with L15:ASW. Intracellular recordings from MNs were made with 10–20 MΩ sharp electrodes filled with 3 M potassium acetate connected to an Axoclamp 2-B amplifier (Molecular Devices). Data acquisition and analyses of resting potential, input resistance, and EPSP amplitude were performed with pCLAMP 8 software (Molecular Devices). Before measurement of EPSPs, MNs were held at −90 mV by passing constant current. Cultures were excluded from further use if pre-treatment measurements of EPSP amplitudes were less than 10 mV, larger than 35 mV, or sufficiently large to trigger an action potential. MNs with resting potentials more positive than −40 mV or input resistances less than 10 MΩ were also excluded. These measurements were repeated 24 h after treatment. The number of samples (n) reported in Results indicates the number of co-cultures. All the above experiments were performed in a blind manner so that the investigator performing the electrophysiology was unaware of the treatment the neurons received.

### Statistical analyses

SigmaPlot version 11 (Systat Software, Inc.) was used to perform all statistical analyses. For immunocytochemistry, SNs were isolated from the same animal in each experimental repetition. Therefore, the paired t-test (between two groups), or repeated measures one-way (RM) ANOVA was used on raw data, followed by the post hoc Student-Newman–Keuls method (SNK) for multiple comparisons analysis^13,14,16,21^. For measuring multiple time points of pRSK after 5-HT (Fig. 3), the Wilcoxon Signed Rank Test with Bonferroni corrections was used for comparison between paired Veh and 5-HT treatment groups, because data from one of the time points failed a normality test. Adjusted p values after Bonferroni corrections were used to represent statistical significance.

For electrophysiological experiments, the amplitudes of the EPSPs, or the numbers of APs for excitability measurements, were assessed before (pre-test) and 24 h after 5-HT treatment (post-test). In contrast to immunofluorescence, these data were normalized for one-way ANOVA analysis, followed by SNK post hoc analysis. Post-test data were normalized to the corresponding measurements made at pre-test. To analyze the changes in resting potentials and input resistance of MNs and SNs caused by BID, we measured the resting potentials and input resistance before (pre-test) and 24 h after vehicle or BID treatment (post-test). Student’s t-test was used to compare the changes [(post-pre)/pre)] in the BID group with those in the vehicle group.

Data from all experiments were presented as means ± SEM, and *P* <0.05 was considered to represent statistical significance.

## Supplementary information

Supplementary Information.

## References

[1] Poirier R (2007). Deletion of the Coffin-Lowry syndrome gene Rsk2 in mice is associated with impaired spatial learning and reduced control of exploratory behavior. Behav. Genet..

[2] Xing J, Ginty DD, Greenberg ME (1996). Coupling of the RAS-MAPK pathway to gene activation by RSK2, a growth factor-regulated CREB kinase. Science.

[3] Xing J, Kornhauser JM, Xia Z, Thiele EA, Greenberg ME (1998). Nerve growth factor activates extracellular signal-regulated kinase and p38 mitogen-activated protein kinase pathways to stimulate CREB serine 133 phosphorylation. Mol. Cell Biol..

[4] Finkbeiner S (1997). CREB: a major mediator of neuronal neurotrophin responses. Neuron.

[5] Choi YH (2011). The extracellular signal-regulated kinase mitogen-activated protein kinase/ribosomal S6 protein kinase 1 cascade phosphorylates cAMP response element-binding protein to induce MUC5B gene expression via D-prostanoid receptor signaling. J. Biol. Chem..

[6] Rawashdeh O, Jilg A, Maronde E, Fahrenkrug J, Stehle JH (2016). Period1 gates the circadian modulation of memory-relevant signaling in mouse hippocampus by regulating the nuclear shuttling of the CREB kinase pP90RSK. J. Neurochem..

[7] Delaunoy JP, Dubos A, Marques Pereira P, Hanauer A (2006). Identification of novel mutations in the RSK2 gene (RPS6KA3) in patients with Coffin-Lowry syndrome. Clin. Genet..

[8] Morice E (2013). Defective synaptic transmission and structure in the dentate gyrus and selective fear memory impairment in the Rsk2 mutant mouse model of Coffin-Lowry syndrome. Neurobiol. Dis..

[9] Lonze BE, Ginty DD (2002). Function and regulation of CREB family transcription factors in the nervous system. Neuron.

[10] Zhang Y (2011). Computational design of enhanced learning protocols. Nat. Neurosci..

[11] Hu JY, Levine A, Sung YJ, Schacher S (2015). cJun and CREB2 in the postsynaptic neuron contribute to persistent long-term facilitation at a behaviorally relevant synapse. J. Neurosci..

[12] Montarolo PG (1986). A critical period for macromolecular synthesis in long-term heterosynaptic facilitation in *Aplysia*. Science.

[13] Liu RY, Cleary LJ, Byrne JH (2011). The requirement for enhanced CREB1 expression in consolidation of long-term synaptic facilitation and long-term excitability in sensory neurons of *Aplysia*. J. Neurosci..

[14] Liu RY, Fioravante D, Shah S, Byrne JH (2008). cAMP response element-binding protein 1 feedback loop is necessary for consolidation of long-term synaptic facilitation in *Aplysia*. J. Neurosci..

[15] Romeo Y, Zhang X, Roux PP (2012). Regulation and function of the RSK family of protein kinases. Biochem. J..

[16] Liu RY (2013). Deficit in long-term synaptic plasticity is rescued by a computationally predicted stimulus protocol. J. Neurosci..

[17] Sapkota GP (2007). BI-D1870 is a specific inhibitor of the p90 RSK (ribosomal S6 kinase) isoforms *in vitro* and *in vivo*. Biochem. J..

[18] Hart AK (2011). Serotonin-mediated synapsin expression is necessary for long-term facilitation of the Aplysia sensorimotor synapse. J. Neurosci..

[19] Zhou L (2015). Rescue of impaired long-term facilitation at sensorimotor synapses of *Aplysia* following siRNA knockdown of CREB1. J. Neurosci..

[20] Enslen H, Tokumitsu H, Soderling TR (1995). Phosphorylation of CREB by CaM-kinase IV activated by CaM-kinase IV kinase. Biochem. Biophys. Res. Commun..

[21] Liu RY, Neveu C, Smolen P, Cleary LJ, Byrne JH (2017). Superior long-term synaptic memory induced by combining dual pharmacological activation of PKA and ERK with an enhanced training protocol. Learn. Mem..

[22] Cleary LJ, Lee WL, Byrne JH (1998). Cellular correlates of long-term sensitization in Aplysia. J. Neurosci..

[23] Beck K (2015). Loss of the Coffin-Lowry syndrome-associated gene RSK2 alters ERK activity, synaptic function and axonal transport in Drosophila motoneurons. Dis. Model. Mech..

[24] Asok A, Leroy F, Rayman JB, Kandel ER (2019). Molecular Mechanisms of the Memory Trace. Trends Neurosci..

[25] Bartsch D, Casadio A, Karl KA, Serodio P, Kandel ER (1998). CREB1 encodes a nuclear activator, a repressor, and a cytoplasmic modulator that form a regulatory unit critical for long-term facilitation. Cell..

[26] Bartsch D (1995). Aplysia CREB2 represses long-term facilitation: relief of repression converts transient facilitation into long-term functional and structural change. Cell..

[27] Lee JA, Kim H, Lee YS, Kaang BK (2003). Overexpression and RNA interference of Ap-cyclic AMP-response element binding protein-2, a repressor of long-term facilitation, in *Aplysia* kurodai sensory-to-motor synapses. Neurosci. Lett..

[28] Philips GT, Ye X, Kopec AM, Carew TJ (2013). MAPK establishes a molecular context that defines effective training patterns for long-term memory formation. J. Neurosci..

[29] Martin KC (1997). MAP kinase translocates into the nucleus of the presynaptic cell and is required for long-term facilitation in *Aplysia*. Neuron.

[30] Lakshminarasimhan H, Coughlin BL, Darr AS, Byrne JH (2017). Characterization and reversal of Doxorubicin-mediated biphasic activation of ERK and persistent excitability in sensory neurons of *Aplysia californica*. Sci. Rep..

[31] Muller U, Carew TJ (1998). Serotonin induces temporally and mechanistically distinct phases of persistent PKA activity in *Aplysia* sensory neurons. Neuron.

[32] Sharma SK (2003). Differential role of mitogen-activated protein kinase in three distinct phases of memory for sensitization in *Aplysia*. J. Neurosci..

[33] Mozzachiodi R, Byrne JH (2010). More than synaptic plasticity: role of nonsynaptic plasticity in learning and memory. Trends Neurosci..

[34] Han JH (2007). Neuronal competition and selection during memory formation. Science.

[35] Yiu AP (2014). Neurons are recruited to a memory trace based on relative neuronal excitability immediately before training. Neuron.

[36] Lisman J, Cooper K, Sehgal M, Silva AJ (2018). Memory formation depends on both synapse-specific modifications of synaptic strength and cell-specific increases in excitability. Nat. Neurosci..

[37] Arthur JS (2004). Mitogen- and stress-activated protein kinase 1 mediates cAMP response element-binding protein phosphorylation and activation by neurotrophins. J. Neurosci..

[38] Rahn EJ, Guzman-Karlsson MC, David Sweatt J (2013). Cellular, molecular, and epigenetic mechanisms in non-associative conditioning: implications for pain and memory. Neurobiol. Learn. Mem..

[39] Walters ET (2018). Nociceptive Biology of Molluscs and Arthropods: Evolutionary Clues About Functions and Mechanisms Potentially Related to Pain. Front. Physiol..

[40] Chin, J., Angers, A., Cleary, L. J., Eskin, A. & Byrne, J. H. Transforming growth factor β1 alters synapsin distribution and modulates synaptic depression in *Aplysia*. *J. Neurosci*. **22**, RC220, 20026363 (2002). 10.1523/JNEUROSCI.22-09-j0004.2002 PMC6758346 11978861

[41] Chin J, Angers A, Cleary LJ, Eskin A, Byrne JH (1999). TGF-β1 in *Aplysia*: role in long-term changes in the excitability of sensory neurons and distribution of TβR-II-like immunoreactivity. Learn. Mem..

[42] Mohamed HA, Yao W, Fioravante D, Smolen PD, Byrne JH (2005). cAMP-response elements in *Aplysia* creb1, creb2, and Ap-uch promoters: implications for feedback loops modulating long term memory. J. Biol. Chem..

