# Supplementary material for: Role of p90 ribosomal S6 kinase in long-term synaptic facilitation and enhanced neuronal excitability
Source: Sci Rep. 2020 Jan 17;10:608. doi: 10.1038/s41598-020-57484-y (PMC6969148; doi:10.1038/s41598-020-57484-y)
Supplement: Supplementary file 1 — Supplementary Information. [file 41598_2020_57484_MOESM1_ESM.pdf]

Title: Role of p90 ribosomal S6 kinase in long-term synaptic facilitation and enhanced neuronal excitability

Authors: Rong-Yu Liu, Yili Zhang, Paul Smolen, Leonard J. Cleary, John H. Byrne

Affiliations:

Department of Neurobiology and Anatomy

W.M. Keck Center for the Neurobiology of Learning and Memory

McGovern Medical School at the University of Texas Health Science Center at Houston

6431 Fannin Street, Suite MSB 7.046

Houston, TX 77030

Correspondence should be addressed to:

John H. Byrne, Ph.D., Department of Neurobiology and Anatomy, W.M. Keck Center for the Neurobiology of Learning and Memory, McGovern Medical School of the University of Texas Health Science Center at Houston, Houston, TX 77030. Tel: 713-500-5602, Fax: 713-500-0623. E-mail: John.H.Byrne@uth.tmc.edu

Supplementary materials

Analogy of Aplysia RSK and mammalian RSK2. Multiple sequence alignments with NCBI Blast showed 73% identity between human p90 RSK2 and *Aplysia* RSK and over 90% at the C-terminal kinase domain (residues 540-670), especially near ERK1/2 phosphorylation sites (Fig. S1), suggesting *Aplysia* p90 RSK may function similarly to mammalian isoforms. We note NCBI Blast predicts a second ribosomal protein S6 kinase Alpha 5 (RPS6Kα5)-like gene (XM_013083681.1 with protein Access ID XP_012939135.1). However, this protein has a substantially different molecular weight (about 65kDa) than p90 RSK, and has NCBI official synonym symbols MSK1; RLPK; MSPK1. We further utilized NCBI Blast to compare the mRNA sequences and no significant similarity was found. We therefore believe it is less likely to be analogous to the p90 RSK2 that is mutated in CLS.

BID alone did not significantly change the resting potential or input resistance of MNs, or of isolated SNs (Fig. S2). In the BID group, the resting potential changed 1.0 ± 2.5 % (Pre-test -52 ± 1.9 mV, post-test -53 ± 1.6 mV, n = 13 MNs) 24 h after BID treatment. While in vehicle-treated group, it changed -3.2 ± 4.0 % (Pre-test -53 ± 1.6 mV, post-test -51 ± 2.3 mV, n = 12 MNs). Student's t-test revealed that the percentages changes in resting potential after BID treatment was not significantly different from vehicle (t_{23} = 1.22, P = 0.23). We also analyzed the effect of BID on input resistance of MNs. In BID group, the input resistance changed 6.3 ± 6.3 % (Pre-test 21 ± 1.6 MΩ, post-test 22 ± 1.3 MΩ, n = 13 MNs) 24 h after BID treatment. While in vehicle-treated group, it changed -8.3 ± 6.0 % (Pre-test 22 ± 2.5 MΩ, post-test 19 ± 1.9 MΩ, n = 12 MNs). Student's t-test revealed that the percentages changes in input resistance after BID treatment was not significantly different from vehicle (t_{23} = 1.88, P = 0.07). Therefore, BID itself did not cause significant changes in both resting membrane potential and input resistance of the MNs. In

addition, we examined the effects of BID on the resting potential and membrane resistance of isolated SNs. In BID group, the resting potential changed 1.7 ± 2.3 % (Pre-test -45 ± 0.8 mV, post-test -46 ± 0.9 mV, n = 15 SNs) 24 h after BID treatment. While in vehicle-treated group, it changed -1.2 ± 2.1% (Pre-test -47 ± 1.1 mV, post-test -47 ± 1.4 mV, n = 16 SNs). Student's t-test revealed that the percentages changes in resting potential after BID treatment was not significantly different from vehicle (t_{29} = 0.91, P = 0.77). We also analyzed the effect of BID on input resistance of SNs. In BID group, the input resistance changed -0.2 ± 6.4 % (Pre-test 71 ± 4.2 MΩ, post-test 70 ± 4.0 MΩ, n = 15 SNs) 24 h after BID treatment. While in vehicle-treated group, it changed -2.6 ± 5.2 % (Pre-test 79 ± 4.1 MΩ, post-test 77 ± 5.0 MΩ, n = 16 SNs). Student's t-test revealed that the percentages changes in input resistance after BID treatment was not significantly different from vehicle (t_{29} = 0.29, P = 0.77). Therefore, BID itself did not cause significant changes in both resting membrane potential and input resistance of the SNs.

Reduction of RSK expression by siRNA (Fig. S3). SNs were injected with RSK siRNA or non-targeting-siRNA (Con-siRNA) and then fixed for immunofluorescence to examine RSK protein levels 96 h after injection. Total RSK levels in SNs injected with RSK-siRNA (n = 5 independent experiments with 24 injected SNs) averaged 28 ± 6% less than those in Con-siRNA injected SNs (n = 5, total 28 SNs) (Figs. 5A1, paired t-test, t_4 = 4.63, P = 0.01), indicating that RSK siRNA injection decreased basal RSK expression.

Supplementary figures

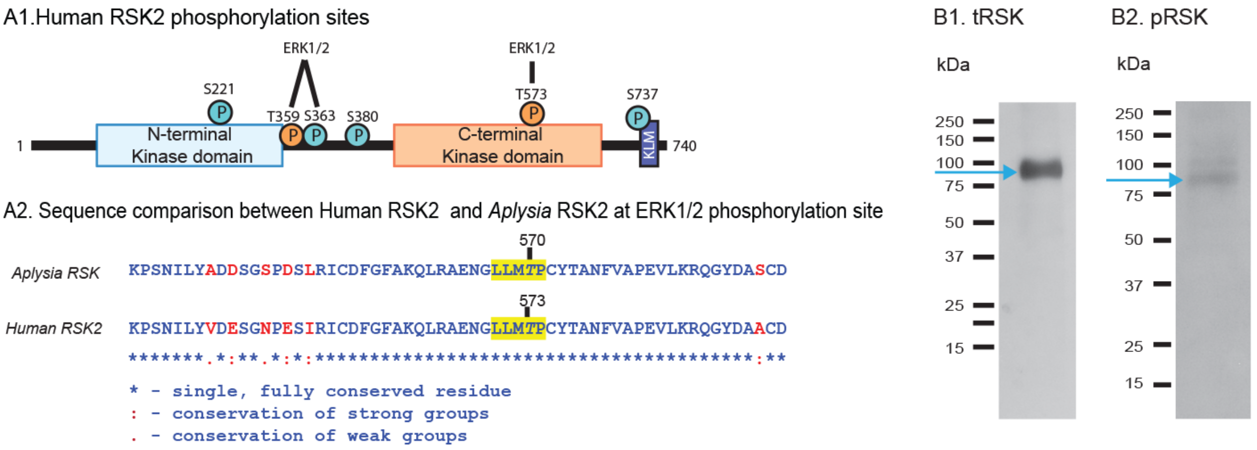

Figure S1. The RSK2 phosphorylation sites and anti-RSK2 antibody validation. A, RSK2 phosphorylation sites. A1, Schematic representation of human RSK, with two phosphorylation sites for ERK 1/2. A2, The position and sequence comparison between human RSK2 and *Aplysia* RSK2 at the ERK1/2 T570/573 phosphorylation site. The peptide sequence surrounding this ERK phosphorylation site in human RSK 2 protein is identical to that in *Aplysia* RSK protein. B, Antibody validation using full-length gels and blots. In each gel, one lane was loaded with protein marker and the other lane was loaded with 30 μg cell lysate from pleural-pedal ganglia. Protein samples were resolved using SDS–-PAGE and then transferred to a nitrocellulose membrane. Membranes were cropped to remove those lanes, which were not loaded protein samples and markers, before antibody incubation. Immunoreactive bands were visualized by ECL. B1, Western blot of total RSK (tRSK). B2, Western blot of phosphorylated RSK (pRSK).

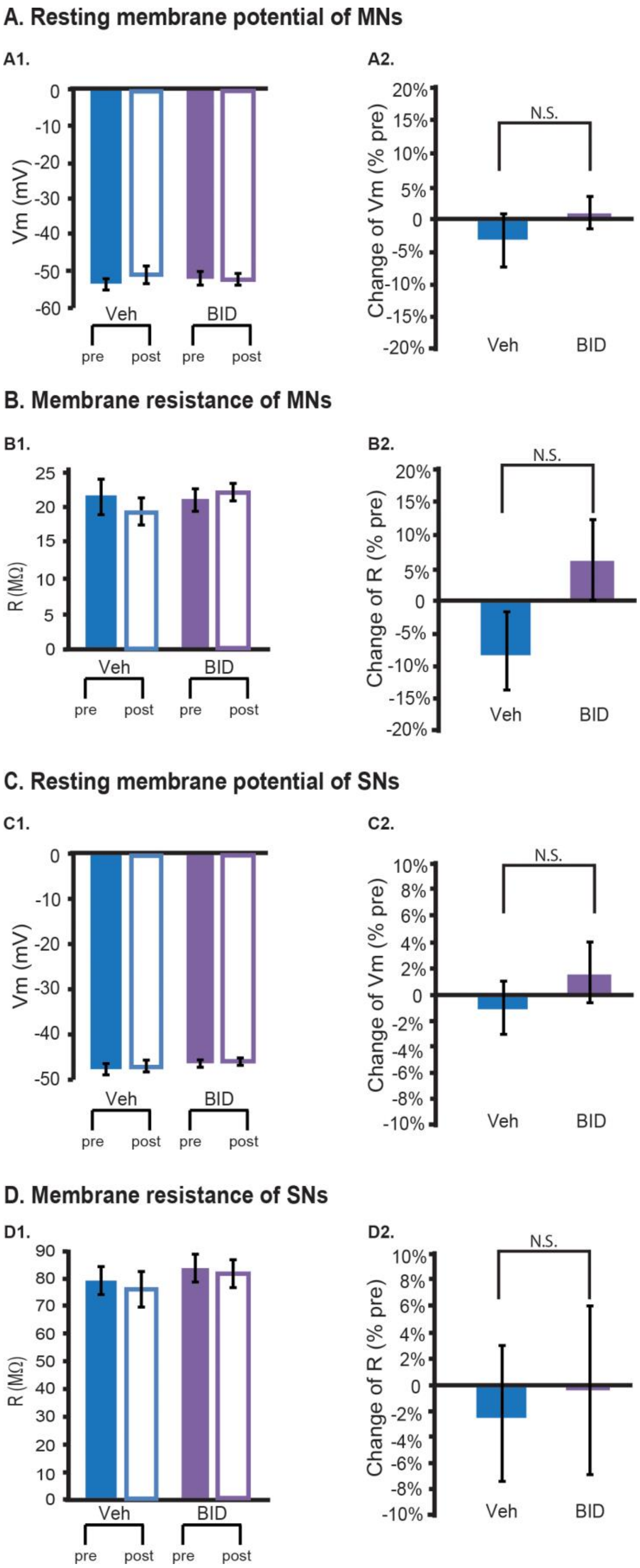
A. Resting membrane potential of MNs
A1.
Vm (mV)
0
-10
-20
-30
-40
-50
-60
Veh
BID
pre
post
pre
post
A2.
Change of Vm (% pre)
20%
15%
10%
5%
0
-5%
-10%
-15%
-20%
N.S.
Veh
BID
B. Membrane resistance of MNs
B1.
R (MΩ)
25
20
15
10
5
0
Veh
BID
pre
post
pre
post
B2.
Change of R (% pre)
20%
15%
10%
5%
0
-5%
-10%
-15%
-20%
N.S.
Veh
BID
C. Resting membrane potential of SNs
C1.
Vm (mV)
0
-10
-20
-30
-40
-50
Veh
BID
pre
post
pre
post
C2.
Change of Vm (% pre)
10%
8%
6%
4%
2%
0
-2%
-4%
-6%
-8%
-10%
N.S.
Veh
BID
D. Membrane resistance of SNs
D1.
R (MΩ)
90
80
70
60
50
40
30
20
10
0
Veh
BID
pre
post
pre
post
D2.
Change of R (% pre)
10%
8%
6%
4%
2%
0
-2%
-4%
-6%
-8%
-10%
N.S.
Veh
BID

Figure S2. BID alone did not significantly change the resting potential or input resistance of MNs, or of isolated SNs. The resting membrane potentials (Vm) and membrane resistance were recorded from the MNs (A and B) or SNs (C and D) before (pre-test) and 24 h after (post-test) treatment with vehicle (Veh) or BID. Left plots show raw data and the right plots showed the percentage changes after Veh or BID treatment. Student's t-test revealed that the percentages changes in either resting membrane potentials or input resistance of MNs and SNs after BID treatment were not significantly different from vehicle (N.S.).

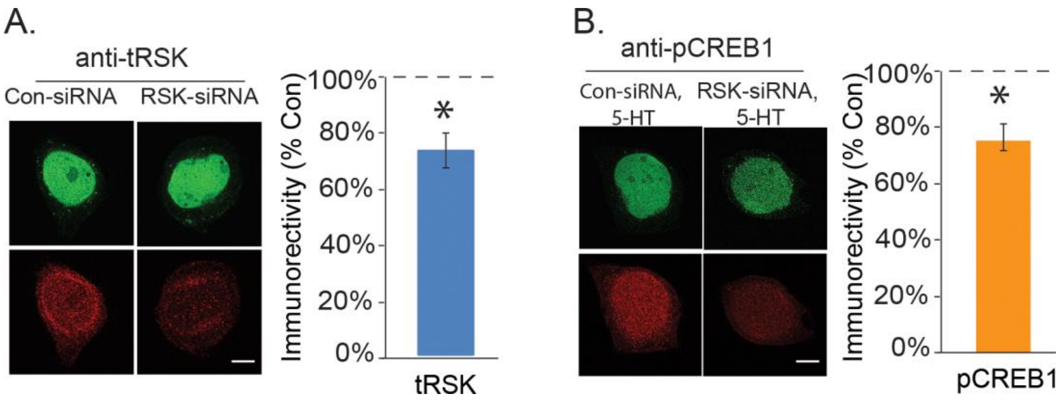

Figure S3. Effects of RSK siRNA on basal RSK protein expression and 5-HT-induced phosphorylation of CREB1. A, Representative confocal images and the summary data of tRSK staining. In the top panel of the confocal images, SNs injected with RSK-siRNA or Con-siRNA were filled at the same time with dye Alexa 488. Bottom panel, the same SNs were immunostained with anti-tRSK. Four days after RSK-siRNA injection, basal RSK protein expression (tRSK, blue bar) was significantly reduced compared to control (n = 5 independent experiments). B, Representative confocal images and the summary data of pCREB1 staining. The 5-HT-induced increase in pCREB1 was also significantly reduced by RSK-siRNA injection (orange bar), compared to Con-siRNA-injected, 5-HT-treated group (n = 5 independent experiments). * for $P < 0.05$ (Paired t-test). Scale bar, 20 μm.
